# Pomegranate Extract Administration Reverses Loss of Motor Coordination and Prevents Oxidative Stress in Cerebellum of Aging Mice

**DOI:** 10.3390/antiox12111991

**Published:** 2023-11-11

**Authors:** David Verdú, Alicia Valls, Ana Díaz, Aitor Carretero, Mar Dromant, Julia Kuligowski, Eva Serna, José Viña

**Affiliations:** 1 Department of Physiology, Faculty of Medicine, University of Valencia, CIBERFES, 46010 Valencia, Spain; david.verdu@uv.es (D.V.); alicia.valls@uv.es (A.V.); acarretero@incliva.es (A.C.); mar.dromant@uv.es (M.D.); jose.vina@uv.es (J.V.); 2 Biomedical Research Institute INCLIVA, University of Valencia, 46010 Valencia, Spain; 3 Central Unit for Research in Medicine (UCIM), University of Valencia, 46010 Valencia, Spain; ana.diaz@uv.es; 4 Neonatal Research Group, Health Research Institute La Fe (IISLaFe), 46026 Valencia, Spain; julia.kuligowski@uv.es

**Keywords:** aging, frailty, pomegranate extract, motor coordination, cerebellum, oxidative stress

## Abstract

The cerebellum is responsible for complex motor functions, like maintaining balance and stance, coordination of voluntary movements, motor learning, and cognitive tasks. During aging, most of these functions deteriorate, which results in falls and accidents. The aim of this work was to elucidate the effect of a standardized pomegranate extract during four months of supplementation in elderly mice to prevent frailty and improve the oxidative state. Male C57Bl/6J eighteen-month-old mice were evaluated for frailty using the “Valencia Score” at pre-supplementation and post-supplementation periods. We analyzed lipid peroxidation in the cerebellum and brain cortex and the glutathione redox status in peripheral blood. In addition, a set of aging-related genes in cerebellum and apoptosis biomarkers was measured via real-time polymerase chain reaction (RT-PCR). Our results showed that pomegranate extract supplementation improved the motor skills of C57Bl/6J aged mice in motor coordination, neuromuscular function, and monthly weight loss, but no changes in grip strength and endurance were found. Furthermore, pomegranate extract reversed the increase in malondialdehyde due to aging in the cerebellum and increased the reduced glutathione/oxidized glutathione (GSH/GSSG) ratio in the blood. Finally, aging and apoptosis biomarkers improved in aged mice supplemented with pomegranate extract in the cerebellum but not in the cerebral cortex.

## 1. Introduction

Aging is defined as the biological process that consists of the progressive deterioration of the organism in apparently healthy individuals [1]. The changes that occur during aging make the individual frailer. Frailty is an age-associated biological syndrome characterized by decreased biological reserves, leading to a decline and deterioration of functional properties at the cellular, tissue, and organ level [2].

During aging, there is cumulative oxidative damage to cellular structures. This can result in mitochondrial oxidative stress and even cell death [3]. Thus, free radicals act as powerful oxidizing agents and cause aging damage by combining with essential molecules, such as DNA and proteins, promoting, among other things, lipid peroxidation, forming malondialdehyde (MDA) as a product, and oxidation of reduced glutathione (GSH) to oxidized glutathione (GSSG). These parameters serve as a reference to understand the oxidative damage that exists in a certain tissue or system [4,5].

Specifically, in aging of the nervous system, a decrease in sensorimotor control and functioning is observed [6]. This leads to loss of motor coordination and eventually to falls. These declines in fine motor control, gait, and balance affect older adults’ ability to perform activities of daily living and maintain their independence. The cerebellum is one of the major areas that control complex motor functions like maintaining balance and stance and coordination of voluntary movements. In the human brain, the cerebellum represents approximately 10% of the total brain volume, and recent studies suggest that the surface area of the cerebellum is 78% of that of the cerebral cortex [7]. The cerebellum seems to be the least studied area of the brain, not only from the point of view of aging but also from a physiological and even anatomical perspective. However, it has a fundamental role in the control of cognitive processes, as well as in the control of motor coordination [8,9].

Nutrition plays a significant role in the aging process, as it can have a profound impact on overall health and well-being as people become older [10,11]. Proper nutrition can help delay the onset of age-related diseases, maintain physical and cognitive function, and improve the quality of life in older adults [12,13]. Therefore, a comprehensive approach that combines healthy eating habits, regular physical activity, stress management, and other healthy lifestyle factors is crucial to promoting healthy aging [14]. Johan Auwerx and Patrick Aebischer identified that urolithin A is able to restore the cell’s ability to recycle the components of defective mitochondria. It is the only known substance that can relaunch the mitochondrial cleaning process. It is a completely natural compound, and its effect is powerful and measurable [15].

In this sense, some studies suggest that pomegranate and its metabolites, such as punicalagin, may also have potential anti-aging properties by reducing oxidative stress and inflammation and improving cell function and longevity [16,17,18]. A recent study shows that pomegranate polyphenol punicalagin is the active molecule that effectively improved neuroinflammation, learning, and memory deficit in natural aging in a 12-month-old mice model and in a D-galactose-induced brain aging model [19]. Although there are basic studies that have shown clear neuroprotective effects of punicalagin in cell lines and mouse models, there are no medical clinical trials so far. Other researchers demonstrated that pomegranate treatment improved cognitive and functional recovery after stroke events and spent less time in the hospital than placebo controls [20]. Furthermore, there are studies on the protection that pomegranate extract exerts against neurodegenerative diseases such as Alzheimer’s disease, Parkinson’s disease, and multiple sclerosis [21,22,23,24,25]. Alzheimer’s is the most prevalent dementia in older people, and aging is their greatest risk factor.

In addition, some clinical and preclinical studies using the same pomegranate extract as ours show metabolic, cardiovascular, and neuronal protection in old individuals [26,27]. More studies on the prevention of natural aging are needed since almost all studies focus on a pathological model.

The aim of this work was to elucidate the effect of pomegranate extract (PE) during 4 months of supplementation in elderly mice from 18 months of age, in which mouse frailty begins, and this supplementation could prevent the frailty associated with natural aging.

## 2. Materials and Methods

### 2.1. Experimental Animals

The animal study was approved by the University of Valencia Ethics Committee for Research and Animal Welfare (license reference: A20201110122413). Animals were housed at the Animal House Core Facility of the University of Valencia.

Male C57BL6 (wild type, WT) eighteen-month mice were assigned to two groups: a control group (only drinking water) and another pomegranate extract (PE) supplementation (150 mg/kg/day) group (solubilized in water).

The PE dosage was adjusted to the liquid intake and to the animal’s body weights every 3 days during 16 weeks’ supplementation. The dose was chosen based on the positive effects found both in metabolic and cardiovascular function in previous clinical studies. As PE was dissolved properly in water, it was used as a vehicle in the control group.

The natural concentrated extract of whole pomegranate (PE) used in this work is Pomanox^®^ P30, obtained through a water-based extraction process and provided by Euromed (Barcelona, Spain).

Pomanox^®^ P30 was prepared according to the European Patent EP1967079 with a technology that utilizes only aqueous solutions, avoiding the use of organic solvents. For manufacturing Pomanox^®^ P30, freshly harvested pomegranate fruits growing in the Spanish Mediterranean area are used. The steps of manufacturing are an extraction of water-soluble compounds, including punicalagins, in aqueous solution, a separation of aqueous solution from pomegranate paste, a step of adsorption chromatography, concentration by nanofiltration, and finally, the pomegranate aqueous extract is turned into a solid form via spray-drying.

According to the manufacturer, Pomanox^®^ P30 (Lot 0100531601) is standardized to >50% total polyphenols and punicalagins, α + β ≥ 30% p/p, and contains α-punicalagin and β-punicalagin (174.4 g/kg and 206.8 g/kg, respectively); galloylglucose, punicalin, and ellagic acid (7.3 g/kg); ellagic acid glucoside (4.2 g/kg); ellagic acid rhamnoside (1.3 g/kg); and the anthocyanins delphinidin-3,5-diglucoside (302.9 mg/kg), cyanidin-3,5-diglucoside (425.3 mg/kg), delphinidin-3-glucoside (75.7 mg/kg), cyanidin-3-glucoside (241.8 mg/kg), and pelargonidin-3-glucoside (99.5 mg/kg). The chemical composition of PE has been characterized by Euromed, and PE polyphenol content is described in Table S1 in Supplementary Material [28]. 

Mice were used in a longitudinal study to evaluate the functional status using the “Valencia Score” and anthropometric determinations before and after supplementation at 18 and 22 months. After mice were euthanized, blood, plasma, and organs were obtained and stored at −80 °C. Samples of WT adult mice (10 months) were used as an aging process control in biomolecular determinations.

### 2.2. Hematological, Biochemical, and Body Composition Parameters

We used the veterinary "hematology element ht5" analyzer (Scil Animal Care Company, Heska group, Viernheim, Germany) located in Central Unit for Research in Medicine (UCIM), Faculty of Medicine, University of Valencia to obtain hematological parameters. Furthermore, we acquired biochemical parameters with the "skyla vb1" (cScil Animal Care Company, Heska group) located in Central Unit for Research in Medicine (UCIM), Faculty of Medicine, University of Valencia using analytical rotors (Critical-care Panel 900-330) in whole EDTA blood from 22-month-old mice without or with supplementation.

The body composition of mice (bone mineral density, lean mass, fat mass, and fat in tissue) was analyzed with simple anesthesia using the method of dual-energy X-ray absorptiometry (DXA) with InAlyzer located in Central Unit for Research in Medicine (UCIM), Faculty of Medicine, University of Valencia (Medikors Inc., Seongnam-si, Republic of Korea).

### 2.3. GSH and GSSG Determination

We determined GSH and GSSG concentrations in blood using the method described by [29].

A total of 10 µL of blood samples was directly added to the papers pre-soaked with 8 µmol of NEM and allowed to derivatize (GSH-NEM formation) at room temperature for 5 min. Then, they were kept at −20 °C until further processing. After, 100 μL of cold PCA solution (4%, *v*/*v*) was added to the precipitate proteins. To extract analytes, tubes with the papers were placed in a thermal mixer for 5 min at 4 °C at 1400 rpm, followed by 5 min of sonication. Finally, samples were centrifuged at 10,000× *g* for 15 min at 4 °C, and 40 μL of supernatants was diluted with 60 μL of H_2_O (0.1% FA, *v*/*v*) and 5 μL of internal standard solution containing isotopically labeled GSH-NEM and GSSG, 100 μmol/L each.

### 2.4. Functional Tests

We evaluated 5 parameters: grip strength (muscular force), rotarod performance test (motor coordination), ladder climbing test (neuromuscular function), treadmill (endurance), and monthly involuntary weight loss (body weight). This score is based on the construct developed by Linda Fried in humans and was termed the “Valencia score” of frailty [30].

#### 2.4.1. Grip Strength Evaluation

To study strength, we use the grip strength test; for this, we use a dynamometer located in Central Unit for Research in Medicine (UCIM), Faculty of Medicine, University of Valencia (Grip Strength Meter, Panlab, Barcelona, Spain) adapted to rodents [31,32]. During the procedure, mice were suspended by the tail, and the force in grams of the front paws was measured. The animals were weighed on a scale to normalize the force to the weight. The animals were held by their tail and placed on the dynamometer, allowing them to grip with the front legs, leaving the animal’s body parallel to the ground. The animals were left held for 20 s, recording the maximum force that the mouse exerted. This procedure was performed 3 times with each animal, leaving 10 min of rest between determinations, and then the maximum force of the 3 tests was recorded.

#### 2.4.2. Motor Coordination Test

To study the motor coordination of mice, rotarod equipment (Panlab, Harvard Apparatus, Holliston, MA, USA) was used [32,33,34,35]. The rotarod is a device made up of a 3 mm diameter bar, separated into 4 spaces for 4 animals, on which the animals are placed. Before the rotarod test, all mice were habituated and pretrained on the rotarod (15 rpm) for 5 min over 3 days. On the test day, the speed of rotation was accelerated gradually from 4 to 40 rpm for 300 s, and the total time spent on the rotarod was recorded. Each mouse was tested over 3 trials, with 15 min intervals between trials.

#### 2.4.3. Endurance and Fatigue Evaluation

To study the fatigue resistance of mice, we used the Treadmill Control LE 8710 equipment (Panlab, Harvard Apparatus) [36,37]. It is a rotating belt that changes the speed of movement, divided into 5 lanes of 38 cm × 5 cm, in which the animals are placed. The distance, time, speed, and number of times the animal receives an electric shock are recorded. For this test, we used the following procedure:

Adaptation of the animals to the test is essential. This is achieved with a 10 min adaptation run. On the day of the test, a race was carried out with a gradual increase in speed. The test ends when it is considered that maximum fatigue has been reached, which is reached when the animal receives 3 electric shocks in 5 s. For the first 4 min, the animals run at 10 cm/s, and from this moment on, the speed is increased by 4 cm/s every 2 min.

#### 2.4.4. Monthly Involuntary Weight Loss (Body Weight)

The body weight of the mice was measured with the precision balance (Gram, Barcelona, Spain, AHZ-300). Three measurements of body weight were assessed on day 0 (before starting supplementation) and every week (until the end of the supplementation program). The mean was calculated for each mouse in each group. Then, to determine individual body weight loss, the percentage of loss was defined for each mouse from 18 months of age to 22 months.

### 2.5. Lipid Peroxidation Determination

Lipid peroxidation determination as malondialdehyde (MDA) levels was determined in cerebellum and brain cortex samples using high-performance liquid chromatography, as described previously [38].

This method is based on the hydrolysis of lipoperoxides and the subsequent formation of an adduct between thiobarbituric acid (TBA) and MDA (TBA-MDA2). This adduct was detected using HPLC in the reverse phase and quantified at 532 nm. The chromatographic technique was performed under isocratic conditions, the mobile phase being a mixture of monopotassium phosphate at 50 mM (pH 6.8) and acetonitrile (70:30). The level of MDA in each sample was divided by the concentration of protein determined using the Lowry method.

### 2.6. Total RNA Extraction and Real-Time Polymerase Chain Reaction (RT-PCR) Studies

Cerebellum and brain cortex were extracted using 300 μL of TRIzol reagent. Integrity quality was performed by Nanodrop 2000 (Agilent, Santa Clara, CA, USA), and the purity was evaluated with the 260/280 ratio. RT-PCR was performed for each of the following genes, using ready-to-use primer and probe sets pre-developed by Applied Biosystems (Foster City, CA, USA, TaqMan Gene Expression Assays) using Quantum Studio v5 (QuantStudioTM Design & Analysis Software v1.4.2), establishing the proper conditions. The housekeeping gene was *Gapdh* (Mm99999915_g1, Applied Biosystems, NM_001289726, NM_008084.4), and the genes studied were Beta-2-Microglobulin (*B2m*) (Mm00437762_m1, NM_009735.3), Caspase 3 (*Casp3*) (Mm01195085_m1, NM_001284409.1, NM_009810.3), Caspase 8 (*Casp8*) (Mm01255716_m1, NM_001080126.2, NM_001277926.2, NM_009812.3), Caspase 9 (*Casp9*) (Mm00516563_m1, NM_001277932.1, NM_015733.5), Lysozyme C (*Lzp-s*) (Mm00657323_m1, NM_013590.4), Cathepsin S (*Ctss*) (Mm00457902_m1, NM_021281.3), Complement 4B (*C4b*) (Mm00437890_m1, NM_009780.2), Complement C1q A Chain (*C1qa*) (Mm00432142_m1, NM_007572.2), and Glial Fibrillary Acidic Protein (*Gfap*) (Mm00546086_m1, NM_010277.3). For the analysis of results, 2^−∆∆Ct^ was used [39].

### 2.7. Statistical Methods

Values are expressed as the mean ± standard deviation (SD) or mean ± standard error (SEM). Normal distribution of the samples was assessed using the Shapiro–Wilk test. To compare two different groups, the unpaired Student’s *t*-test was used, or the Mann–Whitney test in case of a non-normal distribution. To study two independent variables, a two-way analysis of variance (ANOVA) test was used. For the frailty score and its criteria, differences were tested using Pearson’s χ^2^ test. A one-way ANOVA was used to determine differences in gene expression between groups. Statistical analysis was performed using Statistical or GraphPad Prism softwares with a significance level set at *p* < 0.05, and all graphs were represented with GraphPad Prism8 Software version 9.0.0.

## 3. Results

### 3.1. Effect of Pomegranate Extract (PE) on Hematological, Biochemical, and Body Composition

The PE (150 mg/Kg of body weight) does not affect the main hematological and biochemical parameters in the blood of mice (Tables S2 and S3 in Supplementary Material). These values include the blood count, urea, creatinine, and transaminases, among others. Of the 20 parameters measured, the administration of pomegranate extract only affected the lactate concentration. Similarly, the administration of PE does not affect body composition (Table S4 in Supplementary Material).

### 3.2. Effect of Pomegranate Extract (PE) on the Concentration of Glutathione in Mice’s Blood

**Figure 1:**
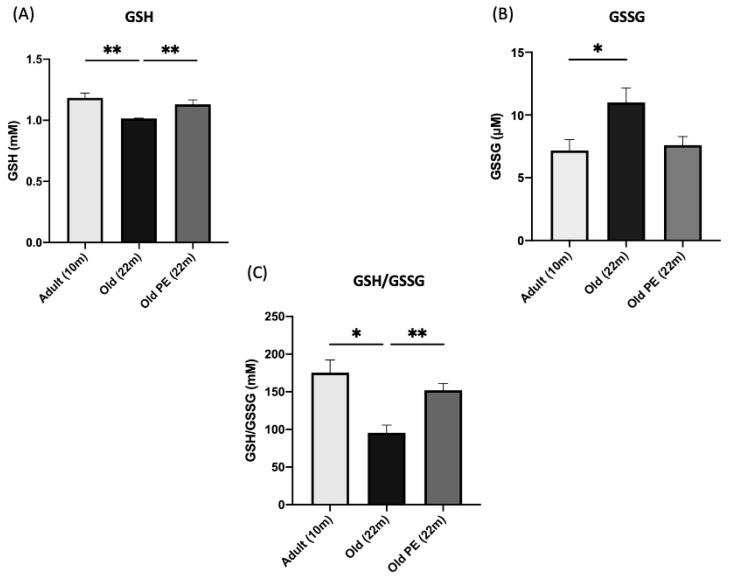
Effect of PE administration on glutathione redox status in mice’s blood. The figure shows (**A**) GSH, (**B**) GSSG, and (**C**) GSH/GSSG levels in blood in mice after 18 months, 22 months, and 22 months of supplementation with PE. Results are expressed as mean ± SEM (*n* = 5–6 per group). * *p* < 0.05, ** *p* < 0.01.

Figure 1 shows the effect of PE administration on glutathione concentration and glutathione redox status in the blood of old mice. Panel A shows that there is a slight decrease in the glutathione value in older animals that is prevented when the animals have been treated with PE. Similarly, glutathione oxidation in the blood of older animals is increased, which is prevented when the animals have taken PE (see panel B). The ratio between reduced and oxidized glutathione is a good index of the redox state of the blood. Panel C shows that the redox ratio of glutathione decreases significantly with age, as expected, and that this decrease is prevented when the animals take PE.

### 3.3. Effect of Pomegranate Extract (PE) on Functional Studies and Body Weight

**Figure 2:**
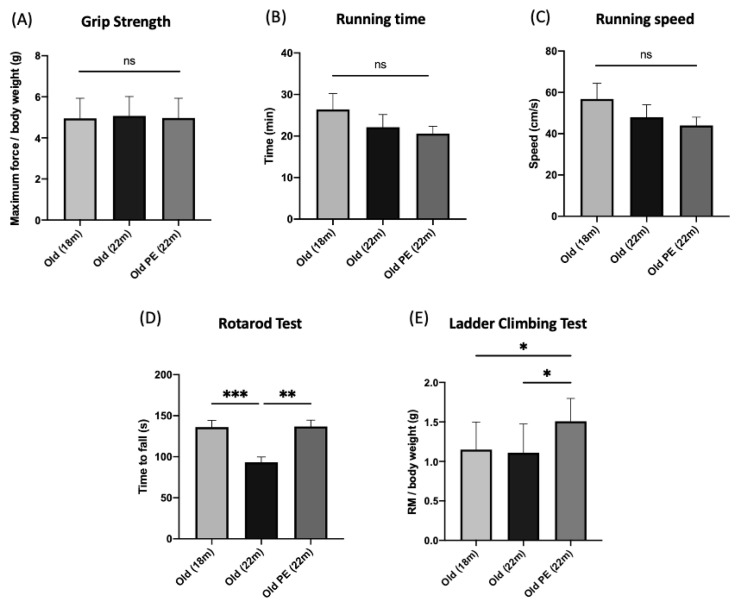
Effect of PE supplementation on functional studies. (**A**) Maximum force on the grip strength test; (**B**) Running time on treadmill; (**C**) Running speed on treadmill; (**D**) Motor coordination on the rotarod test; (**E**) Sensory motor evaluation on the ladder-climbing test. Results are expressed as mean ± SD (*n* = 8–9 per group). * *p* < 0.05, ** *p* < 0.01 and *** *p* < 0.005. (ns: not significant).

Figure 2 shows the effect of PE on functional parameters in aged mice compared with young mice. Panels A, B, and C show that the PE does not produce effects on grip strength, nor on running time or speed. However, there is a positive effect on motor coordination (Panel D, determined by the rotarod; see the Methods section) and on motor strength and coordination determined using the ladder climbing test (Panel E). Therefore, supplementation with PE is favorable to improve functional aspects, especially those related to motor coordination.

**Figure 3:**
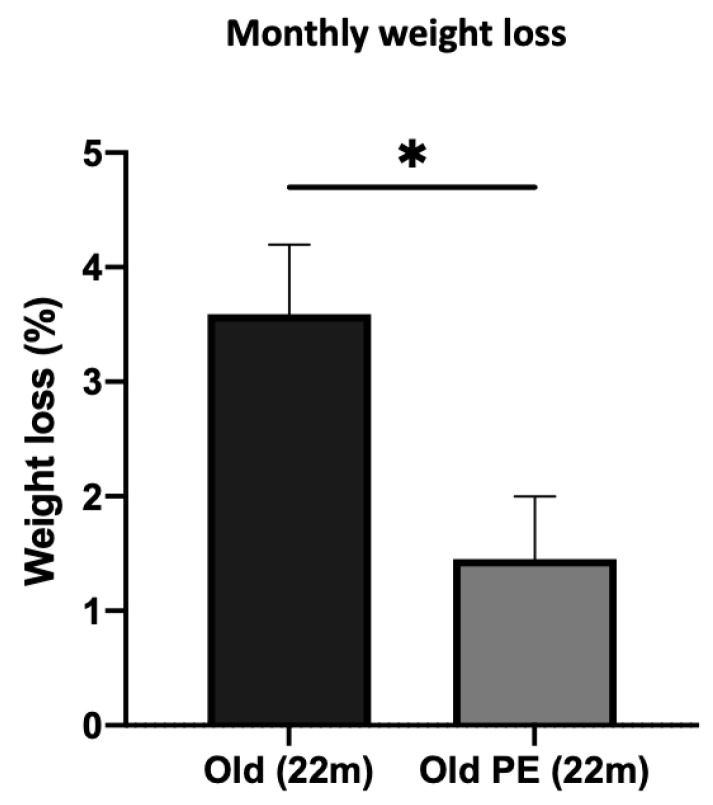
Effect of PE supplementation on weight loss. Data regarding weight loss are expressed as percentage of weight loss after 4 months of supplementation with PE. Results are expressed as mean ± SEM (*n* = 8–9 per group). * *p* < 0.05.

Figure 3 shows that involuntary weight loss after 4-month supplementation is diminished by PE consumption, indicating a better capacity to maintain body weight in aged mice.

### 3.4. Effect of Pomegranate Extract (PE) on Lipid Peroxidation Analyses

In view of these functional results, we analyzed oxidative stress by studying lipid peroxidation in the brain cortex and in the cerebellum because the latter is involved in motor coordination.

**Figure 4:**
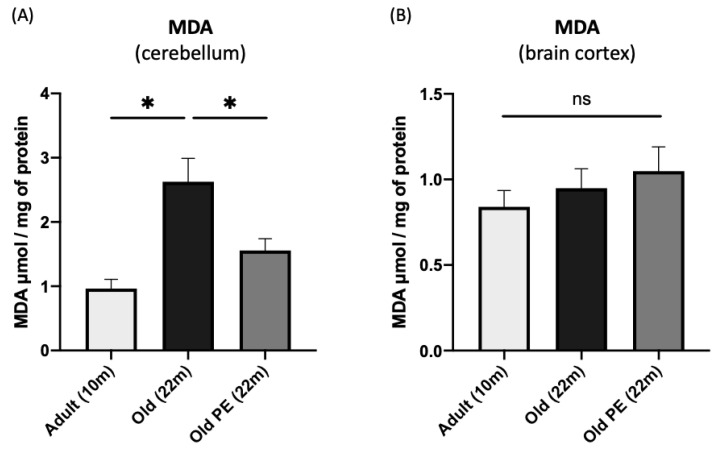
Effect of PE on lipid peroxidation. MDA levels (µmol/mg protein) were measured by HPLC in cerebellum homogenates (**A**) and in brain cortex homogenates (**B**) of mice at 10 and 22 months of age. Results are expressed as mean ± SEM (*n* = 4–7 per group). * *p* < 0.05. (ns: not significant).

Figure 4 shows that PE has a protective effect against lipid peroxidation in the cerebellum but not in the cerebral cortex.

### 3.5. Effect of Pomegranate Extract (PE) on the Expression of Aging Biomarkers in Cerebellum and Brain Cortex

Prolla and his collaborators [40] found a group of genes associated with aging of the cerebellum. These genes are Complement C1q A Chain (*C1qa*), Cathepsin S (*Ctss*), Lysozyme C (*Lzp-s*), Glial Fibrillary Acidic Protein (*Gfap*), and Complement C4B (*C4b*). 

**Figure 5:**
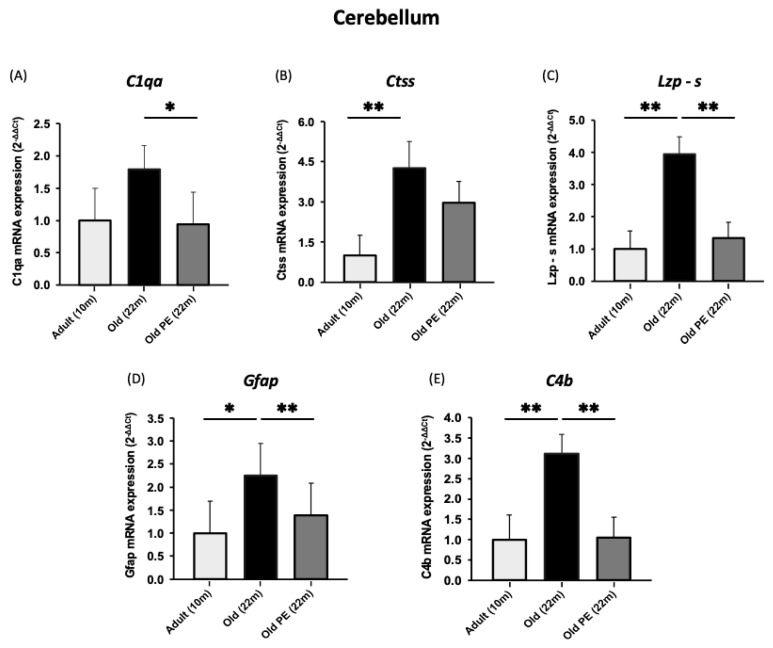
The effect of PE supplementation on the expression of aging biomarkers in cerebellum. (**A**) *C1qa*, (**B**) *Ctss*, (**C**) *Lzp-s*, (**D**) *Gfap*, (**E**) *C4b*. Results are expressed as mean ± SEM (*n* = 7–10 per group). * *p* < 0.05, ** *p* < 0.01.

**Figure 6:**
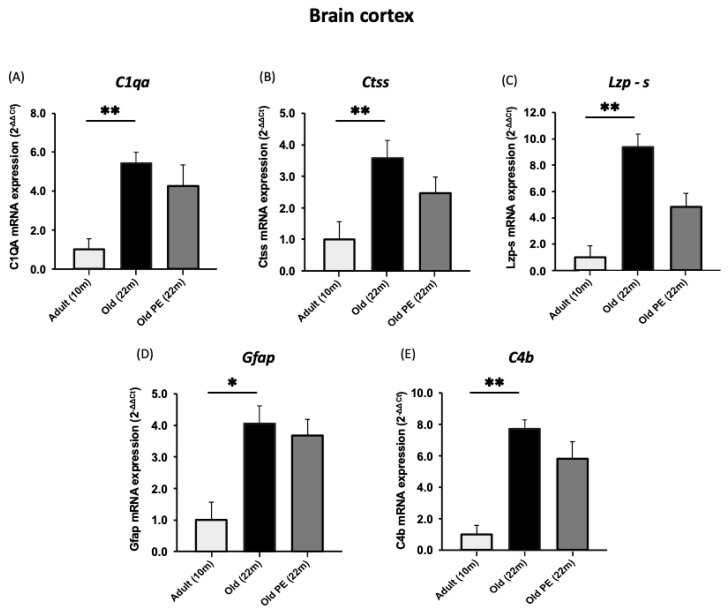
The effect of pomegranate extract supplementation on the expression of aging biomarkers in cortex. (**A**) *C1qa*, (**B**) *Ctss*, (**C**) *Lzp-s*, (**D**) *Gfap*, (**E**) *C4b*. Results are expressed as mean ± SEM (*n* = 5–6 per group). * *p* < 0.05, ** *p* < 0.01.

Figure 5 shows that PE prevents the change in gene expression associated with aging in the cerebellum. The effects are highly significant and contrast with the lack of a protective effect against age-associated changes in the cerebral cortex shown in Figure 6.

**Figure 7:**
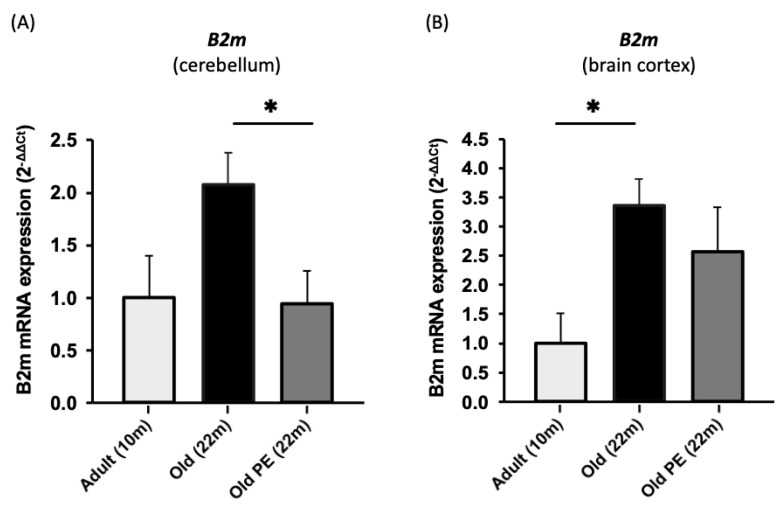
The effect of PE supplementation on the expression of *B2m* in cerebellum (**A**) and brain cortex (**B**). Results are expressed as mean ± SEM (*n* = 5–6 per group). * *p* < 0.05.

We also determined the expression of *B2m* because it has been linked to age-associated neurodegenerative disorders [41]. Figure 7 shows that *B2m* expression increases with age in the cerebellum and brain cortex, but this is only prevented in the cerebellum of the supplemented group. 

### 3.6. Effect of Pomegranate Extract (PE) on the Expression of Apoptosis Biomarkers in Cerebellum and Brain Cortex

**Figure 8:**
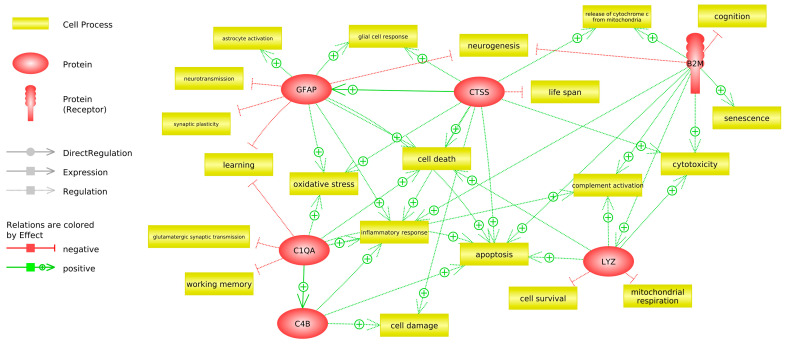
Connections between cell processes and six aging biomarkers. The solid line means expression, and the dashed line means regulation. Relations are colored by effect. Red represents a negative effect, and green represents a positive effect.

An analysis of the relationships between the genes that change with aging and that are protected by the administration of PE showed that the main pathways and cellular processes involved convergence in the control of apoptosis (see Figure 8).

**Figure 9:**
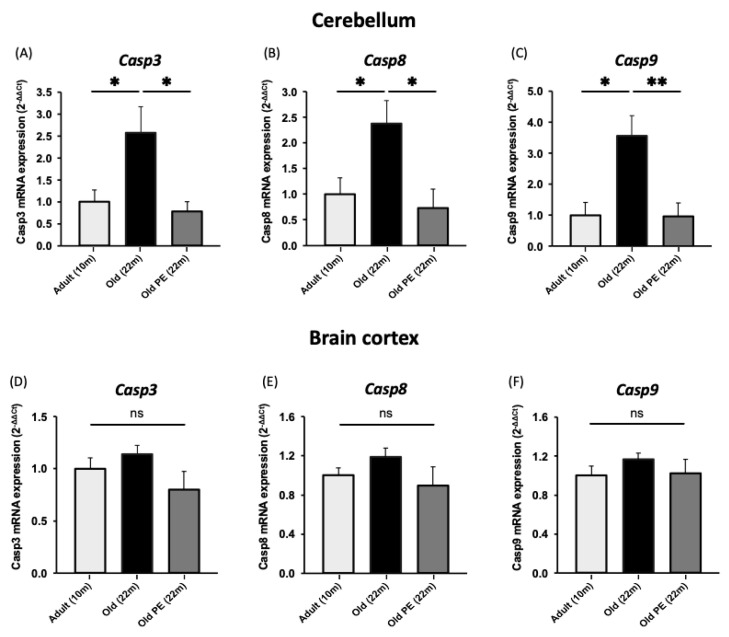
The effect of PE supplementation on the expression of apoptosis biomarkers in cerebellum and cortex. (**A**) *Casp3*, (**B**) *Casp8*, and (**C**) *Casp9* in cerebellum and (**D**) *Casp3*, (**E**) *Casp8*, and (**F**) *Casp9* in cortex. Results are expressed as mean ± SEM (*n* = 6–8 per group). * *p* < 0.05, ** *p* < 0.01. (ns: not significant).

This led us to analyze the expression of genes related to apoptosis, such as *Casp3*, *Casp8*, and *Casp9*, and we observed that they increase with aging and that this increase is protected by the administration of PE in the cerebellum but not in the cortex (see Figure 9).

## 4. Discussion

Older people constitute a key sector of the population. Optimizing nutrition is an essential strategy to promote successful aging [42]. We have found that Pomanox^®^ P30 (standardized pomegranate extract) improves oxidative stress parameters and optimizes gene expression in the cerebellum. Many previous studies have pointed to the importance of pomegranate extracts in promoting improvements ranging from sports performance, blood lipid profile, inflammation, and oxidative stress in various physiological and pathological situations. Endothelial function, and therefore also blood pressure control, also improves in patients treated with pomegranate extracts [43]. Other previous studies in preclinical models demonstrate the capacity of pomegranate and its derivatives to improve lifespan in *C. elegans* [15,44]. Moreover, at the clinical level, pomegranate supplementation, together with protein intake, prevents age-associated adverse events [16].

Frailty is a geriatric syndrome described by Linda Fried and her collaborators [2] that is characterized by slow gait, difficulty getting up, spontaneous weight loss, and feeling unwell. Frailty is a precursor of disability, and therefore, preventing it is a great challenge in geriatric medicine. Frailty was first described in humans. In our research group, we have described a frailty test that we have called the Valencia test [45], extrapolating to animals the changes observed in the tests used in humans. Therefore, our previous studies have made it possible to quantify age-associated frailty in laboratory mice.

Another physiological process implicated in aging and frailty is the involuntary loss of weight. Weight loss and aging are interrelated in several ways. As individuals age, they experience changes in metabolism, body composition, and lifestyle factors that can impact their ability to lose or maintain weight. Metabolism tends to slow down with age, which can make it more challenging to lose weight. This decrease in metabolic rate is partly due to a reduction in muscle mass and a decrease in physical activity levels. As a result, the body burns fewer calories at rest. Our pomegranate extract supplement is able to decrease the loss of weight in aging (see Figure 3).

Falls pose a fundamental risk for dependency and even death in older people [46]. A determining factor in these falls is the decrease in motor coordination associated with age. The fundamental role of the cerebellum in this process has been frequently ignored. Studies by Tom Prolla and his collaborators have shown that the variation of several selected genes with aging is maximum in the cerebellum, even when compared to organs like the heart [47]. We have confirmed these findings (mainly genes that are overexpressed with normal aging), and we have seen that this age-associated change is prevented by the administration of pomegranate extract.

It is now generally accepted that aging is linked to cellular disorganization due to oxidative stress caused by free radicals and other reactive oxygen species (ROS). According to theories published independently by Harman and Gerschman in the 50s of the 20th century, whose central dogma lies in analyzing how, during aerobic metabolism, radical species derived from oxygen are produced incidentally and uncontrollably, macromolecules are irreversibly damaged, damage that accumulates over time, and this results in a gradual loss of homeostatic mechanisms, interference of gene expression patterns, and loss of the cell’s functional capacity that leads to cell aging and death. In addition to the key role of the mitochondrial genome of differentiated cells as the main target of ROS, according to the oxidative stress/mitochondrial injury theory.

Taking all of this together, we have asked ourselves if long-term supplementation with pomegranate extract in 18-month-old mice could prevent a loss of functional capacities and oxidative damage associated with the physiological aging process. After 4 months of supplementation, peripheral blood was extracted and deposited on dry NEM-DBS paper to determine GSH and GSSG levels according to the described method [29]. Supplementation with pomegranate extract keeps GSH and GSSG levels similar to those levels in 10-month-old animals, avoiding the oxidation of GSH to GSSG associated with the aging process that is noted in the control aging group (see Figure 1A–C). These results are in consonance with previous preclinical [48] and clinical [49] studies, where there was an increment in GSH levels in erythrocytes, probably due to the participation of NF-κB and the Nrf2/GSH Axis.

However, the increases that occur in the cerebral cortex with aging are not prevented by the administration of this extract. A limitation of our study is that we have not studied the different areas of the brain because our results regarding phenotyping the animals fed PE led us to changes in coordination and, therefore, to investigate the cerebellum in more detail. The cerebellum, one of the major responsible organs for motor coordination, is exceptional in the large number of major phospholipids that undergo changes mainly by oxidation (with consequential changes in acyl composition) with age, whereas the motor cortex is highly resistant to change [6]. These loss of capacities are, in part, due to the accumulation of oxidative damage, as has been demonstrated by other authors [50,51] where MDA accumulation in the cerebellum and other brain zones by lipid peroxidation reduces motor capacities. For this reason, we have determined lipid peroxidation levels by measuring MDA in the cortex and cerebellum, and we have observed how these levels are lower in animals supplemented for 22 months than in the control group, specifically in cerebellar tissue (see Figure 4A). It is important to note that this reduction is specific to cerebellar tissue and does not appear in the cortex (see Figure 4B).

On the other hand, previous studies have proposed that β2-microglobulin (B2M), a subunit of primary histocompatibility complex class I (MHC I) molecules, regulates behavior, synaptic plasticity, and brain development [52,53]. B2M has been recently considered as a biomarker of Alzheimer’s disease [54,55]. The results from animal studies have emphasized that the local or systemic injection of exogenous B2M in young rats leads to neurodevelopmental and hippocampus-dependent cognitive impairments and that an increase in B2M levels was observed in aged mice, which was verified by cerebrospinal fluid or plasma from healthy populations [41,56]. Our results indicate downregulation of B2M in cerebellar tissue in the supplemented group with respect to the control aged group, which suffers an increment associated with aging (two-fold increment with respect to 10-month-old mice) (see Figure 7).

Finally, we have observed that the genes modulated by the intake of pomegranate extract converge on the apoptosis pathway. These results have been confirmed by analyzing the expression of caspase 3, 8, and 9 (see Figure 8 and Figure 9). We have previously observed that centenarians are also characterized by better control of apoptosis than people with normal aging [57]. The results shown here, together with our studies in centenarians, suggest that apoptosis plays a key role in healthy aging. 

The novelty of this work focuses on the fact that pomegranate extract could be used as a preventive against the deterioration of normal aging. In our study, we have observed for the first time that the cerebellum is the area where aging is first noticed in a model without pathology and where the PE acts efficiently. Therefore, PE could be a good preventative against the risk of falls so common in aging, as well as other functional or biochemical impairments. We have observed changes in levels of oxidative stress, specifically in the cerebellum and without an underlying pathology that other authors observe in the neurodegenerative diseases [58,59]. Furthermore, it is the first study that uses 18-month-old mice, the age at which frailty begins in mice, and PE supplementation reduces part of the functional disabilities and systemic and cerebellar oxidative stress that deteriorate with age.

The main active compounds of our pomegranate extract (see Table S1) are polyphenols and punicalagins. Many articles show that punicalagin has different biological activities, such as in cancer, cardiovascular diseases, liver diseases, and inflammation [60,61,62,63]. Recently, a pharmacological study found that this substance and its polyphenols had significant neuroprotective potential against Alzheimer’s disease (AD), Parkinson’s disease (PD), stroke, and chronic mild stress [20,23,25,64,65]. It is likely that in normal aging, the main beneficial actors are also polyphenols and punicalagins that, when metabolized through the microbiota, generate beneficial active compounds at a systemic level and cross the blood–brain barrier, which would help prevent cerebellar aging and improve the redox status of the elderly.

For this reason, pomegranate extract may be a convenient nutritional intervention to promote healthy aging.

## Data Availability

Data is contained within the article and supplementary material.

## Supplementary Materials

The following supporting information can be downloaded at: https://www.mdpi.com/article/10.3390/antiox12111991/s1, Table S1: Pomegranate polyphenols content from Pomanox^®^ P30; Table S2: Hematological parameters in the blood of old mice with or without supplementation of pomegranate extract; Table S3: Biochemical parameters in the blood of old mice with or without supplementation of pomegranate extract; Table S4: Body composition of old mice with or without supplementation of pomegranate extract.
