# Supplementary material for: Pomegranate Extract Administration Reverses Loss of Motor Coordination and Prevents Oxidative Stress in Cerebellum of Aging Mice
Source: Antioxidants (Basel). 2023 Nov 11;12(11):1991. doi: 10.3390/antiox12111991 (PMC10669012; doi:10.3390/antiox12111991)
Supplement: Supplementary file 1 [file antioxidants-12-01991-s001.zip › antioxidants-2665155-supplementary.pdf]

Table S1. Pomegranate polyphenols content from Pomanox ® P30.

		**Concentration(g/100g)**
**Pomegranate polyphenols**	Pomegranate polyphenols were calculated by adding the sum of punicalagins+ellagic acid+other ellagitanins+anthocyanins	44.2
**Ellagitannins**	Punicalagins	31.6
	Ellagic acid	1.5
	Other ellagitannins (galloylglucose.punicalin. ellagic acid glucoside. ellagic acid rhamnoside)	11.1
**Anthocyanins**	(delphinidin3.5-diglucoside. cyanidin3.5-diglucoside.delphinidin 3-glucoside.cyanidin 3-glucoside.pelargonidin3-glucoside)	<0.1

Table S2. Hematological parameters in the blood of old mice with or without supplementation of pomegranate extract.

**Hematological parameters**	**Old 22 months**		**Old 22 months + PE**		***p*-value**
	**Mean**	**SD**	**Mean**	**SD**	
**WBC (10^9/L)**	4.27	1.47	3.76	1.63	0.38
**Neu # (10^9/L)**	1.52	0.87	1.20	5.03	0.50
**Lym # (10^9/L)**	2.40	1.13	2.00	8.88	0.61
**Eos # (10^9/L)**	0.15	0.08	0.19	3.69	0.69
**Bas # (10^9/L)**	0.01	0.02	0.01	0.06	0.45
**Neu % (%)**	34.72	9.80	33.45	0.29	0.81
**Lym % (%)**	56.10	10.06	50.50	1.27	0.39
**Eos % (%)**	4.53	4.00	5.88	0.11	0.60
**Bas % (%)**	0.38	0.14	0.35	0.01	0.65
**RBC (10^{12}/L)**	7.79	0.26	7.62	0.55	0.49
**HGB (g/dL)**	10.98	0.74	10.63	0.94	0.50
**HCT (%)**	34.52	1.99	33.85	3.91	0.74
**MCV (fL)**	44.27	2.06	44.38	2.84	0.95
**MCH (pg)**	14.10	0.87	13.93	0.38	0.71
**MCHC (g/dL)**	17.08	0.53	32.57	16.29	0.77
**RDW-CV (%)**	16.66	1.04	14.93	1.16	0.07
**PLT (10^9/L)**	731.50	208.47	1592.25	934.74	0.32
**MPV (fL)**	5.46	0.09	5.57	2.78	0.87

PE: pomegranate extract; *n*= 6 Old 22 months' mice and *n*= 4 Supplemented Old 22 months' mice.

Table S3. Biochemical parameters in the blood of old mice with or without supplementation of pomegranate extract.

**Biochemical parameters**	**Old 22 months**		**Old 22 months +PE**		***p*-value**
	**Mean**	**SD**	**Mean**	**SD**	
**Urea (mg/dl)**	58.51	34.18	62.40	14.21	0.83
**Bun**	27.66	15.76	31.42	7.78	0.63
**Alb (g/dl)**	3.15	0.42	3.64	0.47	0.09

**Cpk (mg/dl)**	3982.00	3257.00	7216.00	74445.00	0.92
**Alp (u/l)**	53.85	17.50	56.80	21.39	0.79
**Crea (u/l)**	0.45	0.19	0.53	0.21	0.49
**Alt (u/l)**	32.85	7.05	35.80	15.07	0.65
**Glu (mmol/l)**	163.85	58.93	185.60	61.04	0.54
**Tp (g/dl)**	4.11	1.02	5.02	0.61	0.11
**Lac (mmol/l)**	3.22	1.50	5.22	1.30	0.04*

PE: pomegranate extract; *n*= 7 Old 22 months' mice and *n*= 5 Supplemented Old 22 months' mice. *$p < 0.05$

Table S4. Body composition of old mice with or without supplementation of pomegranate extract.

**Body composition**	**Old 18 months**		**Old 22 months**		**Old 22 months + PE**		***p*- value**		
	**Mean**	**SD**	**Mean**	**SD**	**Mean**	**SD**	**18mo vs 22mo**	**18mo vs 22mo + PE**	**22mo vs 22mo + PE**
**BW**	35.42	3.51	35.05	1.24	35.63	3.59	0.68	0.98	0.74
**%FAT**	23.31	5.19	18.06	3.56	17.23	2.83	0.38	0.17	0.72
**%LEAN**	69.45	19.22	80.1	3.55	80.93	2.55	0.27	0.30	0.70
**BMC (g)**	0.90	0.09	0.79	0.05	0.78	0.09	0.10	0.09	0.94
**BMD (g/cm^2)**	0.08	0.00	0.07	0.01	0.07	0.01	0.07	0.22	0.88
**Bone volume (cm^3)**	0.28	0.03	0.25	0.02	0.19	0.12	0.09	0.23	0.33
**Bone area (cm^2)**	11.91	0.78	11.31	0.27	11.98	1.35	0.12	0.64	0.31

PE: pomegranate extract; mo= months; *n*= 13 Old 18 months' mice. *n*= 5 Old 22 months' mice and *n*= 4 Supplemented Old 22 months' mice.
